# Implementation and clinical impact of an interdisciplinary tool to promote skin integrity after flap surgery in Veterans with spinal cord injury

**DOI:** 10.1080/10790268.2024.2420434

**Published:** 2024-11-20

**Authors:** Byron Eddy, Mary Murphy Kruse, Tina Arneson, Jennifer Hussung, Daniel Greenwood, Crystal Stien, Christie King, Amanda Simone, Gary Goldish, Anjum Kaka, Umar Choudry, Cenk Cayci, Christine M. Olney

**Affiliations:** 1 Spinal Cord Injury and Disorders Center, Minneapolis VA Medical Center, Minneapolis, Minnesota, USA; 2 Department of Infectious Disease, Minneapolis VA Medical Center, Minneapolis, Minnesota, USA; 3 Department of Plastic and Reconstructive Surgery, Minneapolis VA Medical Center, Minneapolis, Minnesota, USA; 4 Department of Plastic and Reconstructive Surgery, University of Minnesota, Minneapolis, Minnesota, USA; 5 Rehabilitation & Engineering Center for Optimizing Veteran Engagement & Reintegration (RECOVER), Minneapolis VA Medical Center, Minneapolis, Minnesota, USA

**Keywords:** Spinal cord injury, SCI, Flap surgery, Pressure injury, Recurrence

## Abstract

**Context/Objective:**

Pressure injury (PrI) recurrence is common among persons with spinal cord injury and disorders (SCI/D) who undergo reconstructive flap surgery for pelvic stage 4 PrI (S4PrI). This paper describes the development and implementation of the Minneapolis Spinal Cord Optimization, Rehabilitation and Empowerment (SCORE), a preoperative interdisciplinary tool for risk assessment and mitigation, and reports its effect on the one-year flap failure rate (significant breakdown).

**Design:**

Retrospective review.

**Setting:**

Minneapolis Veterans Affairs Health Care System (MVAHCS) SCI/D Center, a tertiary care center.

**Participants:**

141 Veterans with SCI/D and pelvic S4PrI underwent 204 interdisciplinary assessments for flap surgery.

**Interventions:**

The Minneapolis SCORE was developed by the MVAHCS SCI/D Center and first implemented in 2012. The eight domains of the tool and continuous improvements in tool implementation for risk assessment and mitigation are described.

**Outcome Measures:**

Per-year incidence rate of flap failures within one year of surgery from 2009 to 2019.

**Results:**

48.3% (28/58) of S4PrI assessments during 2009–2011 (pre-SCORE) led to flap surgery the same year, increasing to 59.6% (87/146) of assessments after SCORE implementation during 2012–2019. The one-year flap failure rate abruptly decreased from 40.5% (15/37) of the 2009–2012 surgeries to 7.7% (6/78) of the 2013–2019 surgeries (P < .0001). Characteristics of patients by time period, operative status, and flap outcome are presented.

**Conclusion:**

After an initial learning curve in tool implementation and subsequent tool refinement, the use of the Minneapolis SCORE before flap surgeries was associated with improved flap integrity at one year. Successful use of the tool requires collaborative problem-solving between the patient and interdisciplinary team.

## Introduction

The prevalence of pressure injury (PrI) in people with spinal cord injury (SCI) for two or more years is 13.1–30.2%, with higher rates among those with more complete SCI and location most often around the pelvis (1–4). Stage 3 or 4 PrI is associated with decreased functional independence and increased mortality (5–8). Garber and Rintala (2) reported that among 57 SCI patients with stage 4 pressure injury (S4PrI), 74.5% did not experience PrI healing and 71.9% were hospitalized for PrI treatment for an average of 150 days over a 3-year observation period. Flap surgical reconstruction for SCI-associated S4PrIs has a complication rate of 21–59.8%, early reoperation rate of 9.7–16%, and ulcer recurrence rate of 12–39% (9–16). To help improve flap outcomes, the Minneapolis Veterans Affairs Health Care System (MVAHCS) Spinal Cord Injury and Disorders (SCI/D) Center implemented a postoperative flap surgery protocol (Supplement 1) in 2009 involving activity restrictions and close monitoring, similar to the 4-week bedrest protocol described by Asanza *et al.* (12). Unfortunately, the average 1-year rate of flap failure (significant breakdown) was 39.3% for surgeries performed at MVAHCS during 2009–2011.

To improve determinations on surgical candidacy and flap surgery outcomes, the MVAHCS SCI/D interdisciplinary team developed a pre-flap surgery risk stratification tool, the Minneapolis Spinal Cord Optimization, Rehabilitation, and Empowerment (SCORE). This tool was first implemented in 2012, and a retrospective analysis on flap surgeries during 2008–2013 in our center found that a higher score on the tool was associated with a higher long-term risk of flap loss and PrI recurrence (17). In addition to risk stratification, the SCI/D team found the SCORE useful for guiding risk mitigation and improving surgical readiness.

The primary purpose of this paper is to describe the process improvement journey involved in Minneapolis SCORE development and implementation, the latter of which evolved over time based on further experience and evidence from the literature. The aim of our project is to help SCI/D patients improve long-term success with flap surgery for S4PrIs with the use of SCORE, as demonstrated by a reduction in the one-year flap failure rate.

## Methods

In 2011, the MVAHCS SCI/D staff conducted a root cause analysis of flap surgery complications that had occurred since 2008. Based on this analysis, staff experience, and previous research, an interdisciplinary team developed the Minneapolis SCORE, which includes eight domains of risk assessment: Medical, Nutritional, Psychological, Physical, Local, Surgical, Social, and Surface (18). Within each domain, three items were scored between 0 and 3 to quantify the relative risk of a negative postoperative outcome, with total score >20 considered high risk. The term “autofail” was applied to some scores of 3 to indicate that mitigation must occur for surgery to be considered.

The Minneapolis SCORE was administered by the interdisciplinary team and implemented in 2012 for pre-flap surgery evaluations in Veterans with SCI/D and pelvic S4PrI at the MVAHCS. If a patient was deemed not appropriate or ready for flap surgery, the team made specific recommendations and helped develop strategies to improve surgical readiness and outcomes.

To present our process improvement activity, within each domain we will describe the scoring process as first implemented in 2012 (Fig. 1). Because we considered the SCORE a living document, we will also describe adaptations to tool implementation during subsequent years based on growing evidence and clinical experience. Supplement 2 shows how adaptations were incorporated into an updated tool after the study period. Figure 1Minneapolis Spinal Cord Optimization, Rehabilitation, and Empowerment (SCORE) tool, 2012 version. Interdisciplinary assessment of candidacy for surgical flap closure of stage 4 pressure injury in patients with spinal cord injury or disorder.

**Figure:**
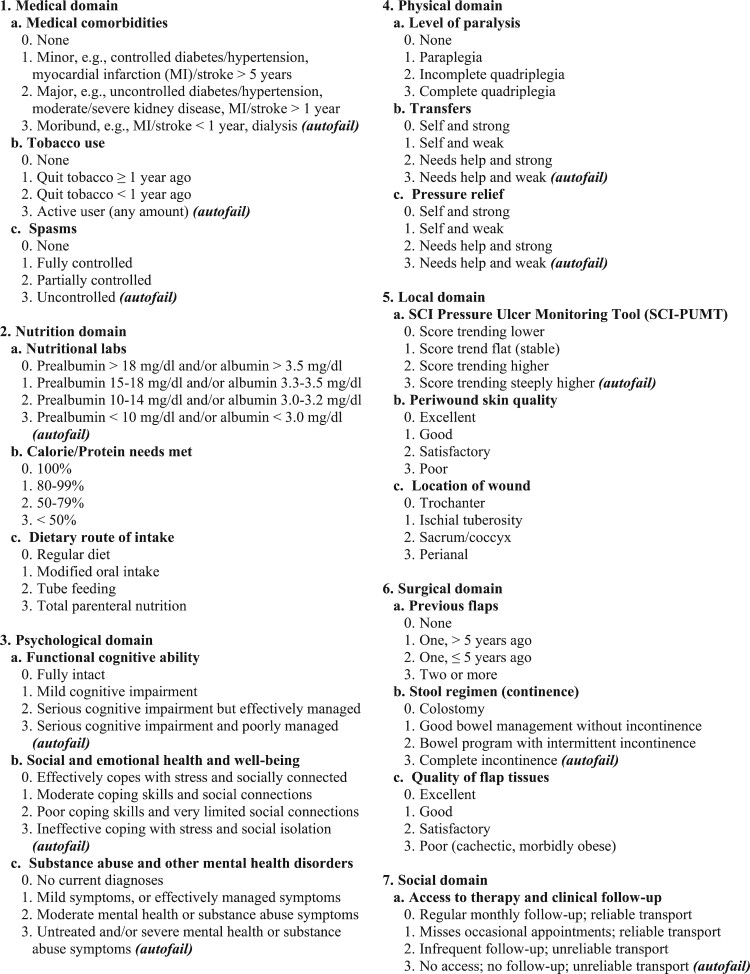

**Figure:**
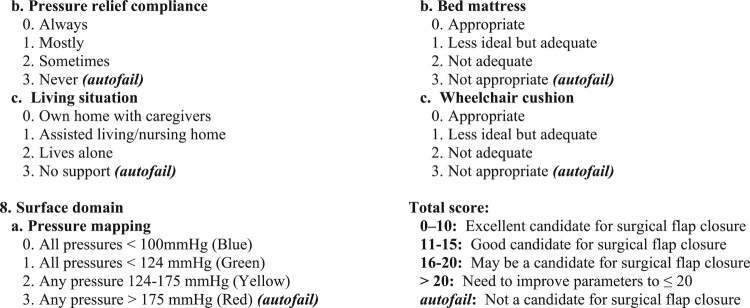

The primary outcome measure, assessed retrospectively, is the annual rate of flap failure within one year of surgery for flaps performed during 2009–2019. Patient characteristics by time period and flap outcome, repeat assessments, reasons for flap failure, and reasons for no flap surgery are also presented. Statistical significance (P < .05) for the primary outcome and patient characteristics was assessed with bivariate chi-square tests or Fisher exact tests for categorical data and two-tailed independent *t*-tests for continuous data.

### Domain 1: medical

The three items in the Medical Domain are *Medical comorbidities*, *Tobacco use*, and *Spasms.* The comorbidity assessment initially focused primarily on cardiovascular diseases, diabetes, and renal failure. Diabetes is particularly known to be associated with postoperative flap surgery complications and PrI recurrence (11, 19–21). Later in the project period, our team shifted to using levels I–IV of the American Society of Anesthesiologists Physical Status Classification System for risk stratification due to its wide use, detailed descriptors, and association with plastic surgery outcomes (22–25).

Tobacco use increases postoperative complications like impaired healing and surgical site infection in multiple types of surgery, including plastic surgery (17, 26–35). PrI recurrence after flap surgery is also higher among tobacco users, with increased risk associated with more years of use (19, 20). Our team offered tobacco cessation interventions to users, and cessation for six weeks was required prior to flap surgery.

Muscle spasms can pose a risk for PrI formation and dehiscence of a recent surgical flap by causing movements, altered positioning, and shear forces (15, 36, 37). The Minneapolis SCORE stratifies spasms according to level of control, specifically regarding spasms that involve the surgical site or induce hip movements or pelvic shear forces. Oral and/or injectable medications for spasticity were given as indicated.

### Domain 2: nutritional

The Nutritional Domain is divided into *Nutritional labs*, *Calorie/protein needs met*, and *Dietary route of intake*. SCORE stratifies albumin and prealbumin levels as relative indicators of nutritional status, though levels can decrease due to factors like inflammation, cardiovascular diseases, cirrhosis, and hypothyroidism, which may also impact flap integrity (38–42). Better surgical outcomes have been reported with prealbumin >15–20 mg/dl and albumin >3–3.5 mg/dl (11, 15, 43–52). Our team primarily tracked prealbumin for short-term changes, which our surgeons required to be ≥18 mg/dl for flap surgery.

Adequate calorie and protein intake serves as the foundation of nutritional assessment for the patient with PrI since malnutrition contributes to one-third of all PrIs (7, 53). This item was assessed according to the percentage intake of recommended calories (30–35 Kcal/kg) and protein (1.25–2 g/kg) for patients with PrI (41, 54, 55). We supported intake through nutrition counseling, meeting food preferences, nutritional support, and micronutrient supplementation (41, 56–61).

The *Dietary route of intake* item stratifies regular diet, modified food, tube feeding, and parenteral nutrition in increasing order of risk. Since dysphagia can worsen in the lying position during post-flap bedrest, routine pre-flap swallow evaluations in that position were added to our assessments during the process improvement period. Our team developed a lower threshold for feeding tube recommendation before flap surgery due to frequent difficulties seen with maintaining consistent and safe intake during bedrest (62–66). Eventually, patient tolerance of tube feeding became scored as zero or one for this item.

### Domain 3: psychological

The following Psychological Domain items reflect the patient’s ability to adhere to postoperative activity restrictions and to engage in the behavioral self-management needed during the months-long recovery at home: *Functional cognitive ability*, *Social and emotional health and well-being*, and *Substance abuse and other mental health disorders*. These indicators were guided by literature on health psychology, psychological factors in disability and rehabilitation, and clinician experiences with the SCI/D population (67–72). Assessments guided appropriate mitigation strategies with willing patients. For example, when mild cognitive impairment was present, the clinical team ensured repetition of instructions and verified the patient was able and willing to perform correct behaviors for successful postoperative recovery. Examples of mitigation for *Social and emotional health and well-being* include reviewing successful coping strategies, addressing barriers to patient engagement with treatment recommendations, encouraging the use of other resources like occupational or recreational therapy, and treatment of associated mental health disorders.

Since tool inception, our team has incorporated the use of standardized assessments in Psychological Domain items to reduce subjective ratings and increase consistency in the protocol.

### Domain 4: physical

The Physical Domain includes the items *Level of paralysis*, *Transfers*, and *Pressure relief*. The SCORE tool initially equated a higher spinal level of SCI/D with increased risk for flap failure based on assumptions about functional status and health maintenance, but further clinical experience and research findings have called this assumption into question (73, 74).

Improper transfers can cause skin injury and affect flap site healing. The descriptors “Self” and “Needs help” in the *Transfers* item signify whether a patient requires assistance, and “Strong” and “Weak” distinguish whether the technique appears safe. Technique considerations include good clearance and control during lateral transfers and the absence of shearing with sliding board transfers or mechanical lift sling placement. Our therapists initially focused on bed/wheelchair transfers, but during the project period, additional transfers (*e.g*. commode, vehicle) were also considered. Further, safety considerations we now include are caregiver reliability, technique consistency, and use of proper equipment (*e.g*. avoid split leg slings during mechanical lifts due to shear). Risk mitigation included education/training, increasing home health hours, and obtaining appropriate equipment.

Knowledge of skin care behaviors, including pressure relief repositioning and skin checking, leads to lower incidence of PrI (75). In the item *Pressure relief*, “Strong” and “Weak” indicate the effectiveness of the pressure relief, which was gauged by the amount of pressure offloaded, the length of pressure relief, and expected tension in the flap region during a pressure relief. Mitigations included education/training on the most appropriate pressure relief method, strength and endurance exercises, and sometimes procurement of a power tilting wheelchair.

### Domain 5: local

The Local Domain assesses the S4PrI in terms of *SCI Pressure Ulcer Monitoring Tool (SCI-PUMT)*, *Periwound skin quality*, and *Location of wound*. Improvements in PrI size, tissue necrosis, and exudate lead to a lower SCI-PUMT score, suggesting that pressure, shear, nonviable tissue, and infection are being adequately managed (76). The interdisciplinary team assessed for and managed potentially contributing problems in accordance with treatment standards (77). During the project period, our wound nurses shifted away from using SCI-PUMT toward more specific monitoring of wound size and color, granulation tissue, bone exposure, necrotic tissue, and signs of infection (78).

*Periwound skin quality* was scored as excellent, good, satisfactory, or poor based on the severity of any periwound concerns such as disruption, rash, erythema, swelling, or maceration. Periwound skin interventions were instituted to promote healthier flap tissue and potentially reduce postoperative complications.

Based on our center’s experience, trochanteric, ischial, sacral/coccygeal, and perianal wound locations were scored in ascending order of risk due to greater post-flap incisional tension and risk of dehiscence during repositioning, plus decreasing patient positioning options. The perianal location has a higher risk of moisture. Risk mitigation involved educating the care team on proper handling during repositioning, which was reinforced by skin markings to indicate where to avoid touching.

### Domain 6: surgical

The Surgical Domain includes the items *Previous flaps*, *Stool regimen (continence)*, and *Quality of flap tissues*. Previous research and our center’s experience suggest that previous flaps may predispose to breakdown of an additional flap in the same body area, and multiple previous flaps limit surgical options (11).

Bowel incontinence can increase the risk of PrI and complications at healing wounds and flap incisions (74, 79, 80). The presence of colostomy was scored as lowest risk since it has been associated with shorter post-flap healing time, fewer ulcer operations, and lower ulcer recurrence rate (81). The SCI/D team helped improve continence as able and sometimes recommended colostomy, particularly for PrIs within two inches of the rectum. We have also started including bladder continence in this assessment due to its effects on skin health.

*Quality of flap tissues* was assessed as excellent, good, satisfactory, or poor based on the amount and thickness of local redundant tissue and presence of scar tissue. These factors affect options for surgical technique, postoperative tissue tension and blood flow, and the capacity for post-flap tissues to withstand pressure. Significant scar tissue, cachexia, and morbid obesity can especially impact incision healing and PrI recurrence (11, 19, 74).

### Domain 7: social

The *Access to therapy and clinical follow-up* item of the Social Domain evaluates the individual’s ability to attend recommended follow-up visits for post-flap monitoring and individualized education, including monthly visits for a year with a wound nurse and at least six months with physical therapy (82). Considerations include access to appropriate and reliable transportation and patient consistency in attending appointments. Mitigation included establishing transportation, education, setting up telehealth services, and procuring appropriate equipment (*e.g*. wheelchair locking systems, cushions for vehicle seats).

The *Pressure relief compliance* item was assessed as per patient, family, or caregiver report of adherence to recommendations for pressure relief repositioning. Our center recommends two minutes of pressure relief after 30 min of sitting for patients without PrI and after 15 min in those with PrI or flap surgery within one year (83). Mitigation in this area included education/training (*e.g*. setting phone alerts) and sometimes procuring a countdown timer.

The *Living situation* item initially equated higher levels of caregiver and social support with lower skin risk, but further experience and research have not consistently supported the caregiver association (3, 16, 84). Risk mitigation in this item included caregiver training, home care securement, home evaluations and modifications, and helping patients find a safer living situation.

### Domain 8: surface

For the Surface Domain, a physical or occupational therapist assessed the appropriateness of the patient’s wheelchair, commode/shower chair, and sleeping surfaces, including interface *Pressure mapping*, which can be predictive of PrI development and healing (58, 85). Using the color-coded XSENSOR® pressure mapping system, peak pressures were stratified into four risk categories.

The patient’s mattresses during hospitalization and at home were assessed for appropriateness based on pressure mapping, body size, and transfer method. In the *Bed mattress* item, a change in mattress was pursued for any score other than “Appropriate.” We initially used an air-fluidized mattress in the early post-flap period, but due to drawbacks with that surface, switched to using a low-air-loss immersion or fluid immersion simulation mattress (86, 87).

For the *Wheelchair cushion* item, the appropriateness of a patient’s seating was assessed according to interface pressures, postural stability and alignment, and patient or caregiver ability to monitor and maintain the cushion. Air cushions require a daily manual check for proper inflation, gel cushions require daily redistribution of gel, and custom foam cushions require consistent and accurate positioning. “Less ideal but adequate” may indicate assistance is needed for cushion management, “Not adequate” means that problems are present, and “Not appropriate” means that the seating is very unsafe. Mitigation included changing the individual’s seated posture or cushion and education/training on proper positioning and cushion maintenance.

## Results

**Figure 2:**
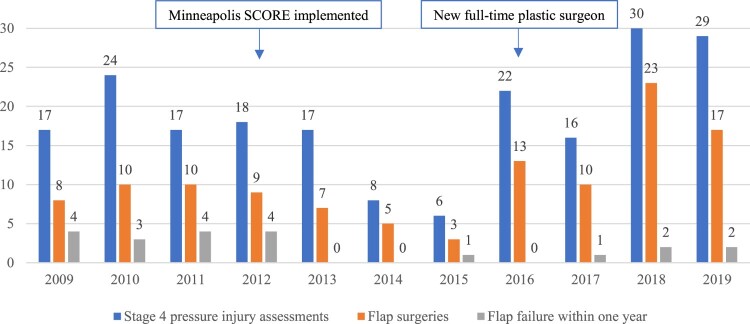
Stage 4 pressure injury assessments and flap surgeries performed during 2009–2019. Numbering is by stage 4 pressure injury assessments, not by unique individuals, counting one assessment per patient per year. Flap failure indicates significant tissue breakdown; these are reported by year of surgery. The Minneapolis Spinal Cord Optimization, Rehabilitation, and Empowerment (SCORE) tool was first implemented in 2012 and a new full-time plastic surgeon began performing flap surgeries in 2016.

Data are presented by S4PrI assessment, counting one assessment per patient per year. Table 1 lists patient characteristics according to pre- and post-SCORE implementation periods, and Table 2 lists characteristics by operative status and flap outcome. The MVAHCS SCI/D Center performed 204 interdisciplinary assessments of pelvic S4PrIs among 141 Veterans during 2009–2019. 48.3% (28/58) of S4PrI assessments during 2009–2011 (pre-SCORE) led to flap surgery, increasing to 59.6% (87/146) of assessments after SCORE implementation during 2012–2019 (Fig. 2). Table 3 provides characteristics of repeat S4PrI assessments of the same patient during the 2009–2019. 68.3% (43/63) of repeat assessments led to flap surgery, and this outcome was significantly more common than no flap surgery among patients who had a previous no-flap determination for the same S4PrI.

**Table 1:** Characteristics of patients assessed for flap surgery, separated by pre- (2009–2011) and post-SCORE (2012–2019) implementation periods.

		2009–2011: pre-SCORE implementation (*n* = 58)	2012–2019: post-SCORE implementation (*n* = 146)	**P** value
Age group (*n*)	18–39	1 (1.7%)	6 (4.1%)	.253
	40–59	21 (36.2%)	40 (27.4%)	
	60–79	30 (51.7%)	92 (63.0%)	
	≥80	6 (10.3%)	8 (5.5%)	
Age (years, mean ± SD)		63.03 ± 11.43	63.40 ± 10.85	.842
Years since SCI/D (mean ± SD)		23.95 ± 15.25	24.16 ± 15.91	.932
Type of SCI/D (*n*)	AIS A tetraplegia	12 (20.7%)	29 (19.9%)	.765
	AIS B tetraplegia	3 (5.2%)	12 (8.2%)	
	AIS C tetraplegia	5 (8.6%)	8 (5.5%)	
	AIS A paraplegia	18 (31.0%)	60 (41.1%)	
	AIS B paraplegia	6 (10.3%)	12 (8.2%)	
	AIS C paraplegia	5 (8.6%)	6 (4.1%)	
	AIS D paraplegia	1 (1.7%)	3 (2.1%)	
	Multiple sclerosis	8 (13.8%)	15 (10.3%)	
** **	Hereditary spastic paraplegia	0	1 (0.7%)	
BMI (kg/m^2^, mean ± SD)		29.36 ± 7.15	26.93 ± 6.61	.022^a^
Diabetes mellitus (*n*)		17 (29.3%)	42 (28.8%)	.939
Tobacco user (*n*)		7 (12.1%)	28 (19.2%)	.224
Location of primary S4PrI (*n*)	Ischial tuberosity	39 (67.2%)	81 (55.5%)	.497
	Sacrum/coccyx	14 (24.1%)	48 (32.9%)	
	Trochanter	4 (6.9%)	14 (9.6%)	
	Perineum	1 (1.7%)	3 (2.1%)	
Multiple flaps to multiple PrIs at once (*n*)		3 (5.2%)	12 (8.2%)	.452
Previous pelvic flap surgery (*n*)	Any previous flap	25 (43.1%)	61 (41.8%)	.863
	Same flap area	15 (25.9%)	34 (23.3%)	.698
	Within past five years	14 (24.1%)	38 (26.0%)	.780
	Two or more flaps	5 (8.6%)	35 (24.0%)	.013^a^
Deaths within three years of PrI assessment (*n*)	Total	20 (34.5%)	49 (33.6%)	.900
** **	Attributed to PrI complications	3 (5.2%)	7 (4.8%)	.910

Notes: SD: standard deviation, SCI/D: spinal cord injury and disorder, AIS: American Spinal Injury Association Impairment Scale, BMI: body mass index, S4PrI: stage 4 pressure injury, SCORE: Spinal Cord Optimization, Rehabilitation, and Empowerment. Numbering is by S4PrI assessments, not by unique individuals. All patients were male except for one female who received flap surgery during 2012–2019. Tobacco user signifies use within six weeks of S4PrI assessment.

^a^ *P* < 0.05.

**Table 2:** Characteristics of patients assessed for flap surgery, by operative status and flap outcome.

		Flap intact at one year (*n* = 94)	Flap failure within one year (*n* = 21)	P value**^a^**	All flaps (*n* = 115)	No flap surgery (*n* = 89)	P value**^b^**
Age group (*n*)	18–39	6 (6.4%)	0	.603	6 (5.2%)	1 (1.1%)	.421
	40–59	27 (28.7%)	6 (28.6%)		33 (28.7%)	28 (31.5%)	
	60–79	56 (59.6%)	13 (61.9%)		69 (60.0%)	53 (59.6%)	
	≥80	5 (5.3%)	2 (9.5%)		7 (6.1%)	7 (7.9%)	
Age (mean ± SD)		62.37 ± 12.00	64.38 ± 9.39	.477	62.74 ± 11.59	63.98 ± 10.18	.429
Years since SCI/D (mean ± SD)		24.37 ± 15.79	22.31 ± 15.34	.591	23.99 ± 15.73	24.23 ± 15.72	.915
Type of SCI/D (*n*)	AIS A tetraplegia	18 (19.1%)	4 (19.0%)	.718	22 (19.1%)	19 (21.3%)	.697
	AIS B tetraplegia	7 (7.4%)	1 (4.8%)		8 (7.0%)	7 (7.9%)	
	AIS C tetraplegia	5 (5.3%)	2 (9.5%)		7 (6.1%)	6 (6.7%)	
	AIS A paraplegia	39 (41.5%)	7 (33.3%)		46 (40.0%)	32 (36.0%)	
	AIS B paraplegia	9 (9.6%)	3 (14.3%)		12 (10.4%)	6 (6.7%)	
	AIS C paraplegia	4 (4.3%)	3 (14.3%)		7 (6.1%)	4 (4.5%)	
	AIS D paraplegia	3 (3.2%)	0		3 (2.6%)	1 (1.1%)	
	Multiple sclerosis	8 (8.5%)	1 (4.8%)		9 (7.8%)	14 (15.7%)	
** **	Hereditary spastic paraplegia	1 (1.1%)	0		1 (0.9%)	0	
BMI (kg/m^2^, mean ± SD)		26.83 ± 5.84	30.25 ± 6.13	.020^c^	27.46 ± 6.04	27.83 ± 7.78	.702
Diabetes mellitus (*n*)		29 (30.9%)	9 (42.9%)	.290	38 (33.0%)	21 (23.6%)	.140
Tobacco user (*n*)		15 (16.0%)	3 (14.3%)	.849	18 (15.7%)	17 (19.1%)	.517
Location of primary S4PrI (*n*)	Ischial tuberosity	50 (53.2%)	10 (47.6%)	.895	60 (52.2%)	60 (67.4%)	.016^c^
	Sacrum/coccyx	34 (36.2%)	9 (42.9%)		43 (37.4%)	19 (21.3%)	
	Trochanter	7 (7.4%)	1 (4.8%)		8 (7.0%)	10 (11.2%)	
	Perineum	3 (3.2%)	1 (4.8%)		4 (3.5%)	0	
Multiple flaps to multiple PrIs at once (*n*)		12 (12.8%)	3 (14.3%)	.852	15 (13.0%)	NA	
Previous pelvic flap surgery (*n*)	Any previous flap	37 (39.4%)	12 (57.1%)	.136	49 (42.6%)	37 (41.6%)	.882
	Same flap area	19 (20.2%)	8 (38.1%)	.081	27 (23.5%)	22 (24.7%)	.837
	Within past five years	24 (25.5%)	7 (33.3%)	.466	31 (27.0%)	21 (23.6%)	.585
	Two or more flaps	18 (19.1%)	6 (28.6%)	.337	24 (20.9%)	16 (18.0%)	.606
Deaths within three years of PrI assessment (*n*)	Total	30 (31.9%)	7 (33.3%)	.900	37 (32.2%)	32 (36.0%)	.571
** **	Attributed to PrI complications	3 (3.2%)	1 (4.8%)	.723	4 (3.5%)	6 (6.7%)	.284

Notes: SD: standard deviation, SCI/D: spinal cord injury and disorder, AIS: American Spinal Injury Association Impairment Scale, BMI: body mass index, S4PrI: stage 4 pressure injury, NA: not applicable. Numbering is by S4PrI assessments, not by unique individuals. All patients were male except for one female who received flap surgery. Tobacco user signifies use within six weeks of S4PrI assessment.

^a^ Comparing ‘Flap intact’ and ‘Flap failure’ groups.

^b^ Comparing ‘All flaps’ and ‘No flap surgery’ groups.

^c^ *P* < 0.05.

**Table 3:** Repeat S4PrI assessments of the same patient during study period, separated by S4PrI location and operative decision for previous and repeat assessments.

	Flap performed after repeat assessment (*n* = 43)	Flap not performed after repeat assessment (*n* = 20)	P value
Previous flap for same location S4PrI (*n*)	13 (30.2%)	5 (25.0%)	.017^a^
Previous flap for different location S4PrI (*n*)	10 (23.3%)	6 (30.0%)	
Previous no-flap determination for same location S4PrI (*n*)	18 (41.9%)	3 (15.0%)	
Previous no-flap determination for different location S4PrI (*n*)	2 (4.7%)	6 (30.0%)	

Notes: S4PrI: stage 4 pressure injury. 32 patients had one repeat assessment; 19 of these led to flap surgery. Three patients had two repeat assessments, four patients had three repeat assessments, two patients had four repeat assessments, and one had five repeat assessments; 24 of these 31 assessments led to flap surgery.

^a^ *P* < 0.05.

The most common flap types were gluteal fasciocutaneous flap, most often used for sacrum/coccyx PrIs, and posterior thigh fasciocutaneous V-Y advancement flap, typically performed for ischial tuberosity PrIs.

**Figure 3:**
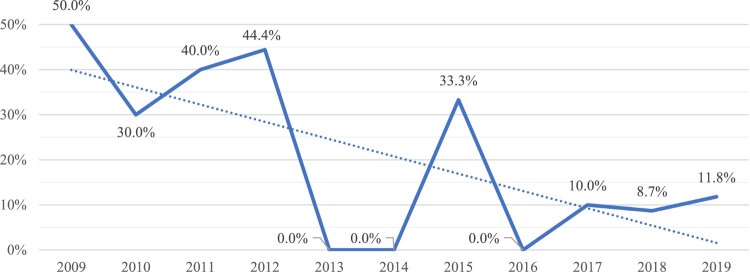
Rate of flap failure (significant breakdown) within one year of flap surgery, reported by year of surgery, with trend line.

The per-year rate of flap failure (significant breakdown) within one year abruptly decreased in 2013 and remained improved in subsequent years except 2015, when one of the three flaps failed (Fig. 3). The average flap failure rate during 2009–2012 was 40.5% (15/37), which decreased to 7.7% (6/78) during 2013–2019 (P < .0001).

During 2018–2019, a SCORE assessment was documented both at the time of initial wound evaluation and around the time of flap surgery. For the 40 flaps performed in this timeframe, the average total score improved from 25.2 to 16.0, with an average interval of 61 days (range 3–233 days) between initial assessment and flap surgery.

The only significant differences in patient characteristics between pre- and post-SCORE implementation groups were that the earlier group had a higher body mass index (BMI) and the latter group more commonly had history of two or more previous pelvic flap surgeries. This difference in BMI was not significant when only considering flap surgeries, *t*(113) = 1.56, P = .122, where pre-SCORE mean BMI was 29.00 ± 5.94 and post-SCORE mean BMI was 26.96 ± 5.99. Comparing all flap and non-flap surgery cases, sacrum/coccyx S4PrI location was significantly more common among flap surgeries and ischial tuberosity S4PrI location was more common among non-flap cases. BMI was significantly higher in flap failure cases than intact flap cases, with BMI ≥35 significantly more common among flap failures (Table 4). A history of previous pelvic flap surgery at any site or the same site was more common among failed flaps than intact flaps, but these differences were not significant. The most common cause of death within three years of assessment among all patients was primary respiratory failure, including pneumonia (21/69, 30.4%), followed by cardiac disease, sepsis, renal failure, cancer, liver disease, stroke, and suicide; the cause was unknown in 10 (14.5%).

**Table 4:** Body mass index of flap surgery patients, by surgical outcome and pre- (2009–2011) and post-SCORE (2012–2019) implementation periods.

		Flap intact at one year (*n* = 94)	Flap failure within one year (*n* = 21)	P value**^a^**	All flaps 2009**–**2011 (*n* = 28)	All flaps 2012**–**2019 (*n* = 87)	P value**^b^**
BMI (*n*)	<18.5	6 (6.4%)	0	.313	0	6 (6.9%)	.226
	18.5–24.9	30 (31.9%)	5 (23.8%)		5 (17.9%)	30 (34.5%)	
	25–29.9	32 (34.0%)	8 (38.1%)		13 (46.4%)	27 (31.0%)	
	30–34.9	18 (19.1%)	3 (14.3%)		6 (21.4%)	15 (17.2%)	
** **	≥35	8 (8.5%)	5 (23.8%)		4 (14.3%)	9 (10.3%)	
BMI ≥35 (*n*)		8 (8.5%)	5 (23.8%)	.045^c^	4 (14.3%)	9 (10.3%)	.567

Notes: SCORE: Spinal Cord Optimization, Rehabilitation, and Empowerment, BMI: Body mass index.

^a^ Comparing ‘Flap intact’ and ‘Flap failure’ groups.

^b^ Comparing ‘All flaps 2009–2011’ and ‘All flaps 2012–2019’ groups.

^c^ *P* < 0.05.

Of the 21 flap failures, ten (47.6%) were primarily due to problems with seating or transfers, five (23.8%) related to nonadherence to postoperative activity recommendations, two (9.5%) resulted from infection, two (9.5%) related to malnutrition, one (4.8%) related to use of an improper mattress, and one (4.8%) resulted from early postoperative venous ischemia of flap tissue. Other contributing factors were severe obesity in two of these, and scar tissue from previous flap surgeries and pelvic obliquity in another.

Among the 89 S4PrI evaluations not leading to flap surgery, 43 (48.3%) did not get surgery because of patient refusal of surgery, postoperative bedrest, or strong interdisciplinary presurgical recommendations; 30 (33.7%) had excessive medical comorbidities, nine (10.1%) were not yet ready for surgery based on interdisciplinary assessments, six (6.7%) lacked surgical options because of prior flap surgeries or size of wounds, and one (1.1%) died before surgery. Comorbidities that prevented surgery were morbid obesity, chronic respiratory failure with recurrent aspiration pneumonia, cirrhosis, renal failure, significant cognitive impairment, severe chronic obstructive pulmonary disease, symptomatic cardiovascular disease, severe malnutrition, and frequent autonomic dysreflexia.

## Discussion

The implementation of a post-flap positioning protocol in 2009 did not initially lead to low 1-year flap failure rates, but the addition of the Minneapolis SCORE in 2012 was significantly correlated with a reduction in flap failures for surgeries performed during 2013–2019. The one-year delay in flap failure reduction post SCORE implementation may have resulted from a learning curve in its optimal use for risk stratification and mitigation. The reduction in S4PrI evaluations and flap surgeries in 2014–2015 was due to lower plastic surgeon availability, and the subsequent increase in these numbers coincided with the hiring of a new full-time plastic surgeon. SCORE helped streamline more comprehensive and consistent S4PrI assessments, leading to less surprises or missed red flags that may affect flap healing. The patient and team gained a better understanding of patient-specific risk factors for complications and strategies to address them.

The exclusion of high-risk patients based on SCORE did not lead to a lower flap surgery rate. The SCORE tool helped guide risk mitigation to improve surgical candidacy and flap site survival, which is supported by the improvement in the total score in the preoperative period, plus high rate of flap surgery completion following repeat S4PrI assessments. The most common reason to not get flap surgery was patient refusal of surgery, postoperative bedrest, or strong interdisciplinary presurgical recommendations, demonstrating that patient acceptance and readiness for change are crucial and often a barrier. The second most common reason for no flap surgery was excessive medical comorbidities, and many of these comorbidities were also the main causes of death within three years of PrI assessment.

Problems with seating or transfers and nonadherence to postoperative activity recommendations accounted for 71.4% of flap failures, highlighting the need for patient engagement in these areas. The other reasons for flap failure also support the need for good infection management, adequate nutrition, and a pressure-distributing mattress. In addition, class II-III obesity (BMI ≥ 35), local scar tissue from previous surgeries, and pelvic obliquity should be cautiously considered in assessments for flap surgery.

The main weakness of the Minneapolis SCORE is that the original tool does not reflect the changes and nuances in tool implementation that our SCI/D interdisciplinary care team adopted over time based on further experience and knowledge of evidence-based practices. In addition, the “autofail” designation for some specific scores may be misinterpreted as non-mitigatable. Our team has now updated a working version of the tool to reflect changes in implementation (Supplement 2). This new version has more scored items, so the original total score risk stratification no longer applies, but our team primarily uses to the tool to support patients and caregivers in effective problem-solving to address specific identified concerns (88).

Another weakness is that tool implementation may be partially limited in some medical settings with fewer clinical resources available. A limitation of the outcomes analysis is that flap failures after one year were not reported, nor were pelvic PrIs that developed after flap surgery in non-flap sites. Postoperative complications not leading to significant flap breakdown were not reported because these were effectively managed without long-term consequences in most cases, as seen in other treatment centers, though they sometimes lengthened the hospital stay (89).

## Conclusion

The Minneapolis SCORE empowers patients and the interdisciplinary health care team to improve the chance of long-term success with flap surgery for pelvic S4PrIs.

## Supplementary Material

Supplement 2 - SCORE tool, updated version.docx

Supplement 1 - Flap Surgery Protocol.doc

SCORE Supplementary material list.docx

## Disclaimer statements

**Funding** This material is the result of work supported with resources and the use of facilities at the Minneapolis VA Health Care System, Minneapolis, MN, USA. One author’s effort on manuscript writing was partly supported by the Paralyzed Veterans of America.

**Disclosure statement** No potential conflict of interest was reported by the author(s).

**Ethics approval** The intervention and data reported in this paper were part of a clinical process improvement project that did not depart from clinical care standards. The MVAHCS institutional review board supported the designation of this project as quality improvement. The authors certify that all applicable institutional and governmental regulations concerning the ethical use of medical records were followed during this study.
