# Supplementary material for: Implementation and clinical impact of an interdisciplinary tool to promote skin integrity after flap surgery in Veterans with spinal cord injury
Source: J Spinal Cord Med. 2024 Nov 20;48(3):415–28. doi: 10.1080/10790268.2024.2420434 (PMC12035953; doi:10.1080/10790268.2024.2420434)
Supplement: Supplement 2 - SCORE tool, updated version.docx [file YSCM_A_2420434_SM1378.docx]

| **Content section** | **SCORE** | **Mitigation** | **Considerations** |
| --- | --- | --- | --- |
| **MEDICAL** | | | |
| 1. **Medical comorbidities**   Levels I-IV of the American Society of Anesthesiologists (ASA) Physical Status Classification System | **0 = Optimal**  ASA I: No medical comorbidities |  |  |
|  | **1 = Acceptable**  ASA II: Mild systemic disease. Examples: well-controlled DM/HTN; mild lung disease; obesity (BMI 30-40) |  |  |
|  | **2 = Possibly acceptable after mitigation**  ASA III: One or more moderate to severe systemic diseases. Examples:  poorly controlled DM/HTN/COPD; morbid obesity (BMI >40); active hepatitis; implanted pacemaker; moderate reduction in ejection fraction; ESRD with dialysis; history (> 3 months) of MI, cardiac stent, stroke, or TIA | Consult medical specialists to optimize management of conditions. |  |
|  | **3 = Unacceptable**  ASA IV: Patient with severe systemic disease that is a constant threat to life. Examples: recent (<3 months) MI, cardiac stent, stroke, or TIA; ongoing cardiac ischemia; severe heart valve dysfunction; severe reduction of ejection fraction; sepsis; DIC; ARDS; ESRD without dialysis |  | Antiplatelet therapy should not be suspended for elective surgery if an arterial stent was placed within the prior 6 months; otherwise, the risk of surgery with or without antiplatelet therapy should be clarified with consultation to the responsible services (eg, cardiology, vascular surgery). |
|  | | | |
| 1. **Tobacco use** | **0 = Optimal**  No prior tobacco use |  |  |
|  | **1 = Acceptable**  Prior tobacco use, but none for at least six weeks | Develop plan to avoid future use | Previous long-term use (> 15-20 years) may increase risk of postoperative pulmonary complications and pressure injury recurrence. |
|  | **2 = Unacceptable/Mitigatable**  Tobacco use within past six weeks and actively engaged with tobacco cessation interventions | Tobacco cessation treatment, including counseling and possibly adjunct medications and/or non-electronic nicotine replacement therapy |  |
|  | **3 = Unacceptable/Not Mitigatable**  Tobacco use within past six weeks and not actively engaged with tobacco cessation interventions | Encourage patient to alert medical team when ready to work toward cessation. |  |
|  | | | |
| Infection management | **0 = Optimal**  No signs of infection |  |  |
|  | **1 = Acceptable**  Infection treatment is ongoing and previous systemic or local signs of infection have resolved. Any necessary debridement has been completed, if applicable. | - Surgical debridement to remove necrotic tissue - Topical wound antimicrobials (eg, Dakin solution) to clear out nonviable tissue and colonization - Cellulitis: 5-14 days antibiotics - Active osteomyelitis: 6 weeks antibiotics, with at least 2 weeks completed prior to flap surgery | - Deep wound cultures, preferably at time of thorough debridement and/or flap surgery. If clinically stable, postpone antibiotics until first deep culture is taken. - Consider imaging for osteomyelitis or deep abscess. - Consider plastic surgery and ID consult |
|  | **2 = Unacceptable/Mitigatable**  Signs of infection are present and patient is accepting of treatment strategies. | Same as above | Same as above |
|  | **3 = Unacceptable/Not Mitigatable**  Signs of infection are present and patient is not accepting of treatment strategies, or there is no acceptable definitive treatment for infection. |  | Same as above |
|  | | | |
| Wound bed preparation/periwound skin quality | **0 = Optimal**  Granular tissue, vascular, moist wound bed, wound contraction, epithelialization, periwound is clear | Conservative wound care; consider wound vacuum-assisted closure (VAC) or electrical stimulation when appropriate. |  |
|  | **1 = Acceptable**  Friable granulation tissue, biofilm, macerated, excess moisture, periwound inflammation | Same as above, plus management of wound or periwound inflammation (eg, infection management, antimicrobial dressing) |  |
|  | **2 = Unacceptable/Mitigatable**  No granulation tissue, fibrinous/slough, bone exposed, malodorous, periwound skin injury, nonviable/necrotic tissue, purulence, denuded periwound skin, cellulitis | Aggressive wound care, including infection management and surgical irrigation and debridement | Consider Plastics consults |
|  | **3 = Unacceptable/Not Mitigatable**  Same as #2, but optimal treatment/debridement is not feasible or declined by patient. | Conservative debridement with wound care products is offered until patient is prepared for surgery. |  |
|  | | | |
| 1. **Prealbumin** | **0 = Optimal**  Prealbumin ≥ 20 mg/dl |  |  |
|  | **1 = Acceptable**  Prealbumin 18-19 mg/dl |  |  |
|  | **2 = Unacceptable/Mitigatable**  Prealbumin < 18 mg/dl and patient accepting treatment strategies | - Optimize protein/calorie intake (see Nutrition domain). - Establish plan for continued nutritional support at home. - Assess for inflammation and potential sources. |  |
|  | **3 = Unacceptable/Not Mitigatable**  Prealbumin < 18 mg/dl and patient declining treatment strategies | Same as above |  |
|  | | | |
| NUTRITION | | | |
| Protein intake | 0 = Optimal100% or greater | Provide education on nutritional needs (protein, calories, fluids, micronutrients) during wound/surgical healing.Adjust food preferences to ensure intake remains adequate.Provide oral nutritional supplements as needed to meet increased needs.Provide feeding assistance and/or adaptive equipment as needed.Monitor weight weekly | Use diet recall/interview to compare protein intake over the past week to needs.Use ideal body weight for predictive equations if the patient is > 120% of calculated ideal weight; otherwise, use actual body weight.Pre-flap and perioperative protein need for stage 3-4 pressure injury: 1.5-2 gm/kgPost-flap (for up to one year) protein need: 1.2-1.5 gm/kgNutritional needs may need to be adjusted as predictive equations not always accurateMonitor renal tolerance of high protein diet. If evidence of excessive intake noted (e.g., hyperglycemia, azotemia, weight gain), adjust nutrition supplements and tube feeding accordingly.Caution providing >2 gm/kg actual body weight; this may lead to dehydration in the elderly. Consult with medical team when protein provision is higher than estimated needs. |
|  | 1 = Acceptable66-99% | Include above plus:Consider feeding tube to supplement diet (see below)*Address any digestive issues that may be limiting intake.Consider registered dietitian (RD) follow-up at discharge. |  |
|  | 2 = Unacceptable/Mitigatable< 66%; accepting of nutritional interventions | Include above plus:- Requires feeding tube- Recommend RD follow-up at discharge |  |
|  | 3 = Unacceptable/Not Mitigatable< 66%; not accepting of nutritional interventions | Ongoing counseling/education needed |  |
| *When considering a feeding tube for a patient, the risks of the procedure should be discussed with the patient and within the patient’s goals of care.Perioperative malnutrition is common due to the catabolic state coupled with inadequate intake and can occur within days in a stressed patient. The postoperative setting for a patient may include pain medication, sedatives, slow return of bowel function, altered mental status and other medical setbacks that make it difficult to consistently meet nutritional demands orally.A feeding tube placed before surgery can be considered as a part of the wound healing treatment plan, as the nutritional demands can often be too high to consume orally on a consistent basis.Depending on baseline aspiration risk, ability to maintain adequate oral intake, presence of malnutrition, and status of wound/flap, the patient will likely not need to keep the feeding tube in place after discharge. | | | |
|  | | | |
| Calorie intake | 0 = Optimal100-150% | Provide education on nutritional needs during wound/surgical healing.Adjust food preferences to ensure intake remains adequate.Provide oral nutritional supplements as needed to meet increased needs.Provide feeding assistance and/or adaptive equipment as indicated.Monitor weight weekly | Use diet recall/interview to compare calorie intake over the past week to needs.Use calorimetry or predictive equations to assess needs.Pre-flap and perioperative calorie needs for stage 3-4 pressure injury: 30-35 kcal/kg or per discretion of RD based on body weight changes.Post-flap calorie needs: 25-30 kcal/kg or per RD to maintain stable weight or modest weight loss with supervision of RD (not to exceed 1 pound per week).Use ideal body weight if the patient is >120% of calculated ideal weight; otherwise, use actual body weight.Degree of weight change can be a measure of caloric excess. Interpret weight with caution, considering changes in fluid status, scales, and equipment. |
|  | 1 = Acceptable66-99%OR> 150% (without significant weight gain) | Include above plus:Consider RD follow-up at discharge  - Recommend multivitamin with minerals supplement  Consider checking for nutrient deficiencies (e.g., zinc, vitamin A, iron)66-99%:Consider feeding tube to supplement diet*Address any digestive issues that may be limiting intake.> 150%:Adjust intake to reduce empty calories.Provide education on medical and equipment implications for weight gain. |  |
|  | 2 = Unacceptable/Mitigatable< 66%OR> 150% PLUS significant, unintended weight gain defined as > 5% in 1 month, > 7.5% in 3 months, > 10% in 6 months, or > 20% in 1 yrThe patient is accepting of nutrition interventions | Include above plus:- Recommend RD follow-up at discharge< 66%:- Requires feeding tube |  |
|  | 3 = Unacceptable/Not MitigatableSame as #2 above, but the patient is not accepting of nutrition interventions | Ongoing counseling/education needed |  |
|  | | | |
| Presence of malnutrition | 0 = Optimal No diagnosis of malnutrition | - See protein and calorie intake sections above | - Assess for malnutrition with American Society for Parenteral and Enteral Nutrition (ASPEN) criteria or another tool. - ASPEN requires 2 of 6 characteristics for a diagnosis of malnutrition: weight loss over time, inadequate energy intake compared with estimated needs, muscle loss, fat loss, fluid accumulation, and diminished grip strength. - Nutrition-focused physical exam may be confounded in the SCI/D population; perform exam above level of SCI if unfamiliar with body habitus. - Etiology of malnutrition may be chronic disease-related, starvation, or acute disease-related. - Resolution of malnutrition should be determined using clinical judgment. - Other assessment tools: Subjective Global Assessment (SGA), Global Leadership Initiative on Malnutrition (GLIM) |
|  | 1 = Acceptable Moderate malnutrition | - Consider feeding tube (depending on cause for malnutrition)  Provide aggressive protein/calorie supplementation until evidence of weight gain or stabilization (about 1 month)  - Address digestive issues that may limit intake - Consider RD follow-up at discharge |  |
|  | 2 = Unacceptable/Mitigatable Severe malnutrition, accepting of interventions | - Recommend feeding tube. May need long-term supplementation to improve nutritional status. - Recommend RD follow-up at discharge |  |
|  | 3 = Unacceptable/Not Mitigatable Severe malnutrition, not accepting of interventions | Further counseling/education needed |  |
|  | | | |
| Aspiration risk | 0 = OptimalTolerates regular diet without risk factors for aspiration AND/OR gastrojejunostomy (GJ) tube in placeIf has regular diet, speech language pathologist (SLP) assessment determined safe and efficient swallowing function. Compensatory strategies or cueing not required. | Provide education on tube feeding administration as needed at discharge or meet criteria for feeding tube removal prior to dischargeProvide feeding assistance or supervision as needed at mealsEat in reverse Trendelenburg if possible, in 30-minute increments; monitor for shearing | SLP assesses for dysphagia while patient is sitting and lying down, in anticipation of flat bedrest after flap surgery.Ongoing assessments may be necessary with patient’s change in condition (e.g., progressive illness, decline in respiratory function).Risk factors for aspiration include but not limited to:TetraplegiaMultiple sclerosisStrokeParkinson’s diseaseSignificant uncontrolled gastroesophageal reflux diseaseAltered mental status/impulsive behavior/dementiaPrior head/neck radiation therapyLung diseaseDependency on others for feeding/drinkingPoor dentition/chewing difficulty |
|  | 1 = AcceptableModified diet or compensatory strategies/cueing required AND/OR risk factors for aspiration AND/OR gastrostomy (G) tube in place | Include above plus:Consider conversion of G-tube to GJ-tube  - SLP to assess need for modified diet, compensatory strategies, cueing, or structured setting |  |
|  | 2 = Unacceptable/MitigatableNot tolerating prescribed diet (i.e., aspiration episodes or high risk of aspiration); feeding tube required and patient is agreeable to placementORNot tolerating feeding tube regimenORTotal parenteral nutrition (PN) | Include above plus:Requires feeding tubeModify enteral nutrition formula, water flushesConsider pharmacologic treatments to support tolerance (antiemetic, prokinetics, antacids, etc.)If on total PN, advance to enteral nutrition with or without supplemental PN when possible.Recommend continued feeding tube and RD follow-up at discharge |  |
|  | 3 = Unacceptable/Not MitigatableNot tolerating prescribed diet; refusal of recommendation for feeding tubeORNot compliant with tube feeding recommendationsORNPO without nutrition support | Ongoing counseling and education needed |  |
|  | | | |
| **SURGICAL OR POSTOPERATIVE CONSIDERATIONS** | | | |
| 1. **Location of pressure injury** | **0 = Trochanter**  Location is easy to offload | Position on a hard and soft turn to opposite of surgery site and back to offload surgery site. |  |
|  | **1 = Ischial tuberosity**  Location has fewer off-loading options, but less pressure than sacral or perianal | Position on hard and soft turns opposite of surgery site. Consider back turns if lateral incision. | Mark with permanent marker below the knee and above the iliac crest to show staff the no-touch zone to protect the flap. |
|  | **2 = Sacrum/coccyx**  Fewer positioning options; more risk of incisional tension, staff pulling, and shear; need to be prone during surgery | Position on hard turns on each side. Avoid back lying. | Be very careful to turn by lower back and knee. Avoid pulling at hip as to not pull on incision. |
|  | **3 = Perianal**  Same issues as sacral location, plus moisture concerns | Position on hard turns on each side. Avoid back lying. Use adductor pillow to maintain air flow to groin. | Most important to avoid moisture buildup in groin and pressure to flap. |
|  | | | |
| 1. Previous flap surgery near current pressure injury | **0 = Optimal**  No previous flaps or surgeries in location of needed flap |  |  |
|  | **1 = Acceptable**  One previous flap at same site |  | Each flap revision increases risk for complications. Scar tissue limits perfusion to flap. |
|  | **2 = Unacceptable/Mitigatable**  Multiple flaps at same site |  | Multiple flaps to same site will limit tissue options and increase risk of complications. |
|  | **3 = Unacceptable/Not mitigatable**  Too many flaps, leaving no technical options left | Conservative wound healing may be best option. |  |
|  | | | |
| Quality of flap tissue | **0 = Optimal**  Redundant tissue, free from scars or skin concerns, good blood supply |  | These factors reduce risk of tension to flap. |
|  | **1 = Acceptable**  Good blood flow, some scarring to work around | Postoperative scar massage can help mitigate risk of complications. |  |
|  | **2 = Unacceptable/Mitigatable**  Limited tissue to use for flap (cachectic, morbid obesity with tight tissue) | - Cachectic: send home with feeding tube  - Morbid obesity: send home to lose weight |  |
|  | **3 = Unacceptable/Not mitigatable**  Repeat flaps with no technical options left | Conservative wound healing may be best option. |  |
|  | | | |
| **SCI/D CONCERNS** | | | |
| 1. **Bladder management** | **0 = Optimal**  Continent or urinary collection system is in place with sustainable plan and maintenance of dry skin. | Reinforce at least daily assessments for pelvic moisture or catheter problems; catheter care. |  |
|  | **1 = Acceptable**  Appropriate bladder management is in place, but with rare or minimal incontinence or catheter bypass, and able to keep pelvic skin dry. | - Same as above - Manage catheter blockages with catheter flushes and/or increasing frequency of catheter changes. - Treat suspected bladder spasms (medications). - If intermittent catheterization is used, ensure adequate cathing frequency and adherence to fluid restriction. |  |
|  | **2 = Unacceptable/Mitigatable**  Bladder management is inappropriate, or incontinence is more than minimal, and patient is willing to follow recommendations. | - Same as above - Recommend indwelling catheter if not present, if bladder management and incontinence are inadequately improved with interventions. - Protect skin appropriately (eg, barrier ointment, moisture-wicking antimicrobial fabric). |  |
|  | **3 = Unacceptable/Not Mitigatable**  Bladder management is inappropriate, or incontinence is more than minimal, and patient is not willing to follow recommendations. | Further education |  |
|  | | | |
| Bowel management | **0 = Optimal**  Colostomy present, or consistently continent of stool with effective bowel program (including while on bedrest) | If colostomy is not present, maintain well-formed stool and watch for factors that may cause loose stool (eg, medications, change to a primarily tube-fed diet). |  |
|  | **1 = Acceptable**  Effective bowel program is in place, but with rare minimal incontinence that causes minimal to no soiling of skin. | - Same as above - Reassess management of bowel program; may consider increased frequency, adjusted timing, or modified method of bowel evacuation. - Closely monitor for further incontinence. - Protect skin as appropriate. |  |
|  | **2 = Unacceptable/Mitigatable**  Occasional incontinence with skin soiling, and patient is willing and able to follow recommendations to establish an effective bowel program or get a colostomy.  Wound proximity to the anus causes difficulty with the surgical procedure being proposed, and patient agrees to colostomy. | - Same as above - Dietary assessment for contributors. - Adjust bowel medications or medications with gastrointestinal side effects. - Recommend colostomy when appropriate. | Stool soiling a fresh flap incision is a risk for major complications. |
|  | **3 = Unacceptable/Not Mitigatable**  Same as #2 above, but patient is not willing or able to follow recommendations. | Further education |  |
|  | | | |
| 1. Spasm management   Assess for spasms in bed and wheelchair. | **0 = Optimal**  Minimal to no spasms in lower body |  |  |
|  | **1 = Acceptable**  Mild to moderate spasms of the lower body that cause <10^o^ of hip motion and do not cause pelvic shear forces or a shift in body position | Oral spasticity medication |  |
|  | **2 = Unacceptable/Mitigatable**  Spasms of the lower body that cause >10^o^ of hip motion and/or cause pelvic shear forces or a shift in body position  **OR**  Spasms or constant tone in muscles to be used in flap surgery | - Oral spasticity medication (increase dose, add additional agents) - Consider chemodenervation and/or neurolysis - Contracture or tendon/muscle release may be considered in some cases |  |
|  | **3 = Unacceptable/Not mitigatable**  Same as #2 above, but unable to adequately treat or patient not accepting of treatment. | Patient education; treatments as appropriate |  |
|  | | | |
| **THERAPY** | | | |
| Equipment: primary mobility device | **0 = Optimal/Not Applicable**  See definition |  | To score Optimal, the following criteria must be met:   1. Surface must provide sufficient pressure reduction and/or clearance of bony prominences 2. Patient must have ability to adequately complete pressure relief on the equipment 3. The equipment must support patient to maintain posture to prevent shearing and friction |
|  | **1 = Acceptable**  Can assume a low level of skin risk to support comfort/function |  |  |
|  | **2 = Unacceptable/Mitigatable**  Moderate to high risk of skin injury; patient agrees to mitigation |  |  |
|  | **3 = Unacceptable/Not Mitigatable**  Declines mitigation |  |  |
| Equipment: shower chair | **0 = Optimal/Not Applicable**  See definition |  |  |
|  | **1 = Acceptable**  Can assume a low level of skin risk to support comfort/function |  |  |
|  | **2 = Unacceptable/Mitigatable**  Moderate to high risk of skin injury; patient agrees to mitigation |  |  |
|  | **3 = Unacceptable/Not Mitigatable**  Declines mitigation |  |  |
| 1. **Equipment: commode chair** | **0 = Optimal/Not Applicable**  See definition |  |  |
|  | **1 = Acceptable**  Can assume a low level of skin risk to support comfort/function |  |  |
|  | **2 = Unacceptable/Mitigatable**  Moderate to high risk of skin injury; patient agrees to mitigation |  |  |
|  | **3 = Unacceptable/Not Mitigatable**  Declines mitigation |  |  |
| Equipment: sleeping surface | **0 = Optimal/Not Applicable**  See definition |  |  |
|  | **1 =Acceptable**  Can assume a low level of skin risk to support comfort/function |  |  |
|  | **2 = Unacceptable/Mitigatable**  Moderate to high risk of skin injury; patient agrees to mitigation |  |  |
|  | **3 = Unacceptable/Not Mitigatable**  Declines mitigation |  |  |
| 1. **Equipment: vehicle seat/cushion** | **0 = Optimal/Not Applicable**  See definition |  |  |
|  | **1 = Acceptable**  Can assume a low level of skin risk to support comfort/function |  |  |
|  | **2 = Unacceptable/Mitigatable**  Moderate to high risk of skin injury; patient agrees to mitigation |  |  |
|  | **3 = Unacceptable/Not Mitigatable**  Declines mitigation |  |  |
|  | | | |
| Safety/adherence: care for equipment (cushions) | **0 = Optimal**  Patient or caregiver follows recommendations accurately and consistently. |  |  |
|  | **1 = Acceptable**  Variability in performance; can assume a low level of risk to support comfort/function |  |  |
|  | **2 = Unacceptable/Mitigatable**  Unsafe; lacking consistent proper technique (shear present); faulty/inappropriate equipment |  |  |
|  | **3 = Unacceptable/Not Mitigatable**  Declines mitigation |  |  |
| Safety/adherence: pressure reliefs (weight shifts) | **0 = Optimal**  Patient or caregiver follows recommendations accurately and consistently. |  |  |
|  | **1 = Acceptable**  Variability in performance; can assume a low level of risk to support comfort/function |  |  |
|  | **2 = Unacceptable/Mitigatable**  Unsafe; lacking consistent proper technique (shear present); faulty/inappropriate equipment |  |  |
|  | **3 = Unacceptable/Not Mitigatable**  Declines mitigation |  |  |
| 1. **Safety/adherence:** **other self-care techniques per training** | **0 = Optimal**  Patient or caregiver follows recommendations accurately and consistently. |  |  |
|  | **1 = Acceptable**  Variability in performance; can assume a low level of risk to support comfort/function |  |  |
|  | **2 = Unacceptable/Mitigatable**  Unsafe; lacking consistent proper technique (shear present); faulty/inappropriate equipment |  |  |
|  | **3 = Unacceptable/Not Mitigatable**  Declines mitigation |  |  |
|  | | | |
| Transfers: primary mobility device | **0 = Optimal/Not Applicable**  See definition |  | When scoring, consider the patient or caregiver’s ability to skillfully, safely, and consistently perform transfers to all relevant surfaces and equipment.  To score Optimal, the following criteria must be consistently met:   1. Ability to complete at all indicated times of day 2. Ability to complete without signs of shear 3. Ability to complete with and without clothing, when applicable |
|  | **1 = Acceptable**  Variability in performance; can assume a low level of risk to support comfort/function |  |  |
|  | **2 = Unacceptable/Mitigatable**  Unsafe; lacking consistent proper technique (shear present); faulty/inappropriate equipment |  |  |
|  | **3 = Unacceptable/Not Mitigatable**  Declines mitigation |  |  |
| Transfers: shower chair | **0 = Optimal/Not Applicable**  See definition |  |  |
|  | **1 = Acceptable**  Variability in performance; can assume a low level of risk to support comfort/function |  |  |
|  | **2 = Unacceptable/Mitigatable**  Unsafe; lacking consistent proper technique (shear present); faulty/inappropriate equipment |  |  |
|  | **3 = Unacceptable/ Not Mitigatable**  Declines mitigation |  |  |
| 1. **Transfers:** c**ommode chair** | **0 = Optimal/ Not Applicable**  See definition |  |  |
|  | **1 = Acceptable**  Variability in performance; can assume a low level of risk to support comfort/function |  |  |
|  | **2 = Unacceptable/Mitigatable**  Unsafe; lacking consistent proper technique (shear present); faulty/inappropriate equipment |  |  |
|  | **3 = Unacceptable/Not Mitigatable**  Declines mitigation |  |  |
| Transfers: sleeping surface | **0 = Optimal/Not Applicable**  See definition |  |  |
|  | **1 = Acceptable**  Variability in performance; can assume a low level of risk to support comfort/function |  |  |
|  | **2 = Unacceptable/Mitigatable**  Unsafe; lacking consistent proper technique (shear present); faulty/inappropriate equipment |  |  |
|  | **3 = Unacceptable/Not Mitigatable**  Declines mitigation |  |  |
| 1. **Transfers:** v**ehicle seat/cushion** | **0 = Optimal/Not Applicable**  See definition |  |  |
|  | **1 = Acceptable**  Variability in performance; can assume a low level of risk to support comfort/function |  |  |
|  | **2 = Unacceptable/Mitigatable**  Unsafe; lacking consistent proper technique (shear present); faulty/inappropriate equipment |  |  |
|  | **3 = Unacceptable/Not Mitigatable**  Declines mitigation |  |  |
|  | | | |
| **PSYCHOLOGICAL** | | | |
| Depression | **0 = Optimal**  No diagnosis of depression or subthreshold symptoms of depression (adjustment disorder is an exception) |  | Current Patient Health Questionnaire-9 (PHQ-9) score < 4 |
|  | **1 = Acceptable**  Past diagnosis of depression with no or subthreshold current symptoms. |  | Current PHQ-9 score < 9 |
|  | **2 = Unacceptable/Mitigatable**  Meets criteria for current diagnosis of depression, being effectively treated [regular mental health (MH) support, medication] or with willingness to engage with treatment | - Psychotherapy (while outpatient or inpatient) - Medication - Engagement in pleasurable activities - Use of smartphone app/independent strategies for mood management - Engagement with Whole Health interventions - Consider referral to specialized care | Current diagnosis of depression per chart or current symptoms in the moderate to severe range for depression (PHQ-9 score > 10) |
|  | **3 = Unacceptable/Not Mitigatable**  Meets criteria for current diagnosis of depression, not being effectively treated and patient unwilling to engage with treatment |  | Current diagnosis of depression per chart or current symptoms in the moderate to severe range for depression (PHQ-9 score > 10) |
|  | | | |
| Anxiety | **0 = Optimal**  No diagnosis of anxiety or subthreshold symptoms of anxiety (adjustment disorder is an exception) |  | Current General Anxiety Disorder-7 (GAD-7) score < 5 |
|  | **1 = Acceptable**  Past diagnosis of anxiety with no or subthreshold current symptoms |  | Current GAD-7 score < 10 |
|  | **2 = Unacceptable/Mitigatable**  Meets criteria for current diagnosis of anxiety, undergoing treatment (regular MH support, medication) or with willingness to engage with treatment | - Psychotherapy - Medication - Engagement in pleasurable activities - Use of smartphone app/independent strategies for mood management - Engagement with Whole Health interventions - Consider referral to specialized care | Current diagnosis of anxiety per chart or current symptoms in the moderate to severe range for anxiety (GAD-7 score ≥ 15) |
|  | **3 = Unacceptable/Not Mitigatable**  Meets criteria for current diagnosis of anxiety**,** not being effectively treated and patient unwilling to engage with treatment |  | Current diagnosis of anxiety per chart or current symptoms in the moderate to severe range for anxiety (GAD-7 score ≥ 15) |
|  | | | |
| Post-traumatic stress disorder (PTSD) | **0 = Optimal**  No diagnosis of PTSD or subthreshold symptoms of PTSD |  | Primary Care PTSD Screen for DSM-5 (PC-PTSD-5) score < 3 |
|  | **1 = Acceptable**  Past diagnosis of PTSD with no or subthreshold current symptoms |  | PC-PTSD-5 score < 3 |
|  | **2 = Unacceptable/Mitigatable**  Meets criteria for current diagnosis of PTSD, undergoing treatment (regular MH support, medication) or with willingness to engage with treatment | - Psychotherapy - Medication - Engagement in pleasurable activities - Use of smartphone app/independent strategies for mood management - Engagement with Whole Health interventions - Consider referral to specialized care | Current diagnosis of PTSD per chart or current symptoms in the moderate to severe range for PTSD (PC-PTSD-5 score > 3) |
|  | **3 = Unacceptable/Not Mitigatable**  Meets criteria for current diagnosis of PTSD**,** not being effectively treated and patient unwilling to engage with treatment |  | Current diagnosis of PTSD per chart or current symptoms in the moderate to severe range for PTSD (PC-PTSD-5 score > 3) |
|  | | | |
| 1. **Serious mental illness** | **0 = Optimal**  No diagnosis of serious mental illness (eg, bipolar disorder, schizophrenia) |  |  |
|  | **1 = Acceptable**  Past diagnosis of serious mental illness with no active mania, delusions, or hallucinations |  |  |
|  | **2 = Unacceptable/Mitigatable**  Current diagnosis of serious mental illness, undergoing treatment (regular MH support, medication) or with willingness to engage with treatment | - Psychotherapy  - Medication  - Consider referral to specialized care |  |
|  | **3 = Unacceptable/Not Mitigatable**  Current diagnosis of serious mental illness, not being effectively treated and patient unwilling to engage with treatment |  |  |
|  | | | |
| Suicide risk May screen with Columbia-Suicide Severity Rating Scale (C-SSRS) | **0 = Optimal**  No current/past suicidal ideation, no past attempts |  |  |
|  | **1 = Acceptable**  Suicidal ideation (active or passive) with plan/intent (> 1 year ago) or passive suicidal ideation without intent/plan (< 1 year ago); past suicide attempts acceptable if > 10 years ago | - Offer mental health support - Provide information on Veterans Crisis Line and other resources - Consider establishing a safety plan |  |
|  | **2 = Unacceptable/Mitigatable**  Current active suicidal ideation (excluding acute high risk) with willingness to accept treatment; attempt(s) within past 10 years | - Same as above, but ensure safety plan is complete - Consider referral to specialized care |  |
|  | **3 = Unacceptable/Not Mitigatable**  Current active suicidal ideation without willingness to accept treatment; attempt(s) within past 10 years |  |  |
|  | | | |
| Cognition | **0 = Optimal**  No cognitive concerns |  | One of the following:   - Neuropsychology testing with no diagnosis in past 3 years and no new cognitive concerns - Blessed Orientation-Memory-Concentration Test (BOMC) score <6 |
|  | **1 = Acceptable**  Some concerns for cognition; should not interfere with ability to adhere to protocol |  | One of the following:   - Neuropsychology testing with diagnosis of Mild cognitive impairment/Mild neurocognitive disorder in past 3 years without new cognitive concerns - BOMC score 7-10 |
|  | **2 = Unacceptable/Mitigatable**  Cognitive concerns that may interfere with adherence to protocol, but are able to be mitigated | Implement compensatory strategies:   - Self-cueing - Phone reminders - Physical reminders around environment - Use of GPS when traveling - Planner/organizer - Other cognitive rehabilitation strategies - Inclusion of caregiver in treatment - Caregiver or healthcare team support for implementation of compensatory strategies - Consider referral to specialized care | One of the following:   - Neuropsychology testing with diagnosis of Major cognitive impairment/Major neurocognitive disorder - BOMC score > 10   **AND**   - Use of effective compensatory strategies or willingness to do so |
|  | **3 = Unacceptable/Not Mitigatable**  Cognitive concerns that may interfere with adherence to protocol, not currently able to be mitigated |  | One of the following:   - Neuropsychology testing with diagnosis of Major cognitive impairment/Major neurocognitive disorder - BOMC score > 10   **AND**   - Unable or unwilling to incorporate effective compensatory strategies |
|  | | | |
| 1. **Alcohol** | **0 = Optimal**  No current alcohol use (past use okay if abstinent at least 1 year) |  | Alcohol Use Disorders Identification Test (AUDIT) score 0 |
|  | **1 = Acceptable**  Past alcohol use with < 1 year of abstinence or current low risk use as defined by AUDIT |  | AUDIT score 1-7 |
|  | **2 = Unacceptable/Mitigatable**  Current problematic alcohol use as defined by AUDIT and willing to moderate use | - Psychotherapy - Attendance at recovery group - Peer support - Consider referral to specialized care | AUDIT score ≥ 8 |
|  | **3 = Unacceptable/Not Mitigatable**  Current problematic alcohol use as defined by AUDIT and not willing to moderate use |  | AUDIT score ≥ 8 |
|  | | | |
| 1. **Drug use** | **0 = Optimal**  No current drug use (past use okay if abstinent at least 1 year) |  | Drug Abuse Screening Test (DAST-10) score 0 |
|  | **1 = Acceptable**  Past drug use with < 1 year of abstinence or current low risk substance use as defined by DAST-10 |  | DAST-10 score 1-2 |
|  | **2 = Unacceptable/Mitigatable**  Current problematic drug use as defined by DAST-10 and willing to moderate use | - Psychotherapy - Attendance at recovery group - Peer support - Consider referral to specialized care | DAST-10 score ≥ 3 |
|  | **3 = Unacceptable/Not Mitigatable**  Current problematic drug use as defined by DAST-10 and not willing to moderate use |  | DAST-10 score ≥ 3 |
|  | | | |
| **SOCIAL SUPPORT AND MOTIVATION** | | | |
| Social support Emotional support may be provided by a family member, friend, spiritual leader, MH professional, or other. | **0 = Optimal**  Has at least one person to talk to for emotional support, and willing to reach out for support |  |  |
|  | **1 = Acceptable**  Has at least one person to talk to for emotional support, not currently willing to reach out, but open to increasing willingness to ask for support |  |  |
|  | **2 = Unacceptable/Mitigatable**  Doesn’t currently have anyone to talk to for emotional support, but willing to figure out who might serve in that role | - Psychotherapy - Identify support people - Reach out to support people - Peer support - Consider referral to chaplain or other professional support person |  |
|  | **3 = Unacceptable/Not Mitigatable**  Doesn’t currently have anyone to talk to for emotional support and not willing to identify someone, or has someone but unwilling to reach out or increase willingness to ask for support |  |  |
|  | | | |
| 1. Access to vehicle/transportation and medical care | **0 = Optimal/ Not Applicable**  See definition |  | To score optimal, patient must have reliable video telehealth access OR all three of the following criteria must be met:   1. Consistent and reliable access to transportation 2. Can effectively perform pressure relief during travel 3. Lives within a practical traveling distance of accessible medical care |
|  | **1 = Acceptable**  Can assume a low level of skin risk to support comfort/function |  |  |
|  | **2 = Unacceptable/Mitigatable**  Does not meet criteria for optimal, but is working toward a solution | - Social worker may assist with community transportation options or alternate housing. - Physical therapist can help establish method of pressure relief during travel. |  |
|  | **3 = Unacceptable/Not Mitigatable**  No form of transportation (personal or public) available; unable to access specialized health care in person or with technology |  |  |
|  | | | |
| Coping | **0 = Optimal**   - Has coping strategies; willing to use them; open to additional support - 1 = yes; 2 = yes; 4 = yes |  | Assessment questions:   1. Do you know strategies to help you cope with things like anxiety, pain, frustration, poor sleep, etc.? If so, what are those strategies? 2. Are you willing to use these strategies during the post-flap recovery period? 3. Are you open to learning coping strategies or working on increasing your willingness to use them? 4. Are you willing to accept support from those around you (e.g., family, care team) to encourage your use of coping strategies? |
|  | **1 = Acceptable**   - Has coping strategies; not willing to use them; open to increasing willingness and accept support - 1 = yes; 2 = no; 3 = yes; 4 = yes |  |  |
|  | **2 = Unacceptable/Mitigatable**   - Doesn’t have coping strategies; willing to develop them and accept support - 1 = no; 3 = yes; 4 = yes | - Psychoeducation for coping strategies - Practice coping strategies in psychotherapy (acceptance and commitment therapy, cognitive behavioral therapy, etc.) - Peer support |  |
|  | **3= Unacceptable/Not Mitigatable**   - Doesn’t have coping strategies and unwilling to learn, or has coping strategies and unwilling to use them or accept support - Any of the following: - 1 = no; 3 = no - 1 = yes; 2 = no; 3 = no - 1 = yes; 2 = no; 3 = yes; 4 = no |  |  |
|  | | | |
| 1. **Values/Motivation**   See Values and Motivation Questionnaire below | **0 = Optimal**  Strong motivation for flap surgery |  | Net score > 11 |
|  | **1 = Acceptable**  Acceptable motivation for flap surgery |  | Net score 1-10 |
|  | **2 = Unacceptable/Mitigatable**  Motivation not currently adequate for flap surgery, but is open to discussing ways to increase motivation | - Motivational interviewing - Psychotherapy - Family intervention/inclusion - Flap education | Both of the following:   - Net score ≤ 0 - Importance > 5 on at least one “no” |
|  | **3= Unacceptable/Not Mitigatable**  Motivation not currently adequate for flap surgery, and not open to discussing ways to increase motivation |  | Both of the following:   - Net score ≤ 0 - Importance < 5 on all “nos” |
| **Values and Motivation Questionnaire** Adapted from the Valued Living Questionnaire and used with permission from Kelly Wilson. Reference: Wilson KG, DuFrene T. Mindfulness for two: an acceptance and commitment therapy approach to mindfulness in psychotherapy. Oakland (CA): New Harbinger; 2009. 256 p.  Patient instructions: Since we know that the flap surgery and recovery process is long and requires commitment, we want to be sure that you feel it is worth it to you. We’ll spend the next few minutes talking about what is important in your life and thinking about how flap surgery might impact those things.   1. Let’s start with your personal relationships – things like romantic relationships, family, or friends. Can you think of something specific in that category that is important to you? 2. Now think about how important that is to you, using a 0 to 10 scale – 0 being not at all important, and 10 being the absolute most important thing. 3. How successful or able do you feel you are to pursue this value right now, while having a wound? Let’s use a 0 to 10 scale again – 0 being not at all successful or able, and 10 being completely successful and able (i.e., the wound does not interfere at all). 4. How do you imagine your success or ability might change if you go through with flap surgery? Again, using a 0 to 10 scale, what is your expected success or ability after the flap? 5. Thinking about all these things together, do you feel that the flap is worth it for this reason?   Repeat questions with each domain.   \| Domain \| Specific Value \| Importance  (0-10) \| Current success/  ability (0-10) \| Expected success/  ability post-flap (0-10) \| Is the flap worth it for this reason? \| Weighted value \| \| --- \| --- \| --- \| --- \| --- \| --- \| --- \| \| Personal relationships (e.g., family, friends) \|  \|  \|  \|  \|  \|  \| \| Employment and/or education/training \|  \|  \|  \|  \|  \|  \| \| Recreation and/or community involvement \|  \|  \|  \|  \|  \|  \| \| Spirituality \|  \|  \|  \|  \|  \|  \| \| Health/physical well-being \|  \|  \|  \|  \|  \|  \| \| Net Score \|  \|  \|  \|  \|  \|  \|   Scoring: Calculate the weighted value in each domain by doing the following:   - If the flap is deemed worth it by the individual for that reason, the weighted value is a *positive* number of the importance rating (e.g., if the importance on personal relationships is a 10 and the flap is deemed worth it for that reason, the weighted value would be 10). - If the flap is deemed not worth it for that reason, the weighted value is a *negative* number of the importance rating (e.g., if the importance on personal relationships is 10 and the flap is NOT worth it for that reason, the weighted value would be -10). - Net score is calculated by taking a sum of all five weighted values. | | | |
|  | | | |

| **Content Section** | **SCORE** | | | | **Comments/Mitigation** |
| --- | --- | --- | --- | --- | --- |
|  | **Initial** | **Pre-Operative** | **Post-Operative** | **6-month Follow-Up** |  |
|  | **Date:** | **Date:** | **Date:** | **Date:** | **Date and mitigation strategies implemented (if applicable) and other rationale for particular score** |
| **Medical** | | | | | |
| 1. Medical comorbidities |  |  |  |  |  |
|  |  |  |  |  |  |
|  |  |  |  |  |  |
|  |  |  |  |  |  |
| 1. Tobacco use |  |  |  |  |  |
|  |  |  |  |  |  |
|  |  |  |  |  |  |
|  |  |  |  |  |  |
| 1. Infection management |  |  |  |  |  |
|  |  |  |  |  |  |
|  |  |  |  |  |  |
|  |  |  |  |  |  |
| 1. Wound bed preparation/   periwound skin quality |  |  |  |  |  |
|  |  |  |  |  |  |
|  |  |  |  |  |  |
|  |  |  |  |  |  |
| 1. Prealbumin |  |  |  |  |  |
|  |  |  |  |  |  |
|  |  |  |  |  |  |
|  |  |  |  |  |  |
| **TOTAL: /15** |  |  |  |  |  |
| **Nutrition** | | | | | |
| 1. Protein intake |  |  |  |  |  |
|  |  |  |  |  |  |
|  |  |  |  |  |  |
|  |  |  |  |  |  |
| 1. Calorie intake |  |  |  |  |  |
|  |  |  |  |  |  |
|  |  |  |  |  |  |
|  |  |  |  |  |  |
| 1. Presence of malnutrition |  |  |  |  |  |
|  |  |  |  |  |  |
|  |  |  |  |  |  |
|  |  |  |  |  |  |
| 1. Aspiration risk |  |  |  |  |  |
|  |  |  |  |  |  |
|  |  |  |  |  |  |
|  |  |  |  |  |  |
| **TOTAL: /12** |  |  |  |  |  |
| **Surgical or Postoperative Considerations** | | | | | |
| 1. Location of pressure injury |  |  |  |  |  |
|  |  |  |  |  |  |
|  |  |  |  |  |  |
|  |  |  |  |  |  |
| 1. Previous flap surgery near current pressure injury |  |  |  |  |  |
|  |  |  |  |  |  |
|  |  |  |  |  |  |
|  |  |  |  |  |  |
| 1. Quality of flap tissue |  |  |  |  |  |
|  |  |  |  |  |  |
|  |  |  |  |  |  |
|  |  |  |  |  |  |
| **TOTAL: /9** |  |  |  |  |  |
| **SCI/D Concerns** | | | | | |
| 1. Bladder management |  |  |  |  |  |
|  |  |  |  |  |  |
|  |  |  |  |  |  |
|  |  |  |  |  |  |
| 1. Bowel management |  |  |  |  |  |
|  |  |  |  |  |  |
|  |  |  |  |  |  |
|  |  |  |  |  |  |
| 1. Spasm management |  |  |  |  |  |
|  |  |  |  |  |  |
|  |  |  |  |  |  |
|  |  |  |  |  |  |
| **TOTAL: /9** |  |  |  |  |  |
| **Therapy** | | | | | |
| 1. Equipment: primary mobility device |  |  |  |  |  |
|  |  |  |  |  |  |
|  |  |  |  |  |  |
|  |  |  |  |  |  |
| 1. Equipment: shower chair |  |  |  |  |  |
|  |  |  |  |  |  |
|  |  |  |  |  |  |
|  |  |  |  |  |  |
| 1. Equipment: commode chair |  |  |  |  |  |
|  |  |  |  |  |  |
|  |  |  |  |  |  |
|  |  |  |  |  |  |
| 1. Equipment: sleeping surface |  |  |  |  |  |
|  |  |  |  |  |  |
|  |  |  |  |  |  |
|  |  |  |  |  |  |
| 1. Equipment: vehicle seat/cushion |  |  |  |  |  |
|  |  |  |  |  |  |
|  |  |  |  |  |  |
|  |  |  |  |  |  |
| 1. Safety/adherence: care for equipment (cushions) |  |  |  |  |  |
|  |  |  |  |  |  |
|  |  |  |  |  |  |
|  |  |  |  |  |  |
| 1. Safety/adherence: pressure reliefs (weight shifts) |  |  |  |  |  |
|  |  |  |  |  |  |
|  |  |  |  |  |  |
|  |  |  |  |  |  |
| 1. Safety/adherence: other self-care techniques per training |  |  |  |  |  |
|  |  |  |  |  |  |
|  |  |  |  |  |  |
|  |  |  |  |  |  |
| 1. Transfers: primary mobility device |  |  |  |  |  |
|  |  |  |  |  |  |
|  |  |  |  |  |  |
|  |  |  |  |  |  |
| 1. Transfers: shower chair |  |  |  |  |  |
|  |  |  |  |  |  |
|  |  |  |  |  |  |
|  |  |  |  |  |  |
| 1. Transfers: commode chair |  |  |  |  |  |
|  |  |  |  |  |  |
|  |  |  |  |  |  |
|  |  |  |  |  |  |
| 1. Transfers: sleeping surface |  |  |  |  |  |
|  |  |  |  |  |  |
|  |  |  |  |  |  |
|  |  |  |  |  |  |
| 1. Transfers: vehicle seat/cushion |  |  |  |  |  |
|  |  |  |  |  |  |
|  |  |  |  |  |  |
|  |  |  |  |  |  |
| **TOTAL: /39** |  |  |  |  |  |
| **Psychological** | | | | | |
| 1. Depression |  |  |  |  |  |
|  |  |  |  |  |  |
|  |  |  |  |  |  |
|  |  |  |  |  |  |
| 1. Anxiety |  |  |  |  |  |
|  |  |  |  |  |  |
|  |  |  |  |  |  |
|  |  |  |  |  |  |
| 1. Post-traumatic stress disorder |  |  |  |  |  |
|  |  |  |  |  |  |
|  |  |  |  |  |  |
|  |  |  |  |  |  |
| 1. Serious mental illness |  |  |  |  |  |
|  |  |  |  |  |  |
|  |  |  |  |  |  |
|  |  |  |  |  |  |
| 1. Suicide risk |  |  |  |  |  |
|  |  |  |  |  |  |
|  |  |  |  |  |  |
|  |  |  |  |  |  |
| 1. Cognition |  |  |  |  |  |
|  |  |  |  |  |  |
|  |  |  |  |  |  |
|  |  |  |  |  |  |
| 1. Alcohol |  |  |  |  |  |
|  |  |  |  |  |  |
|  |  |  |  |  |  |
|  |  |  |  |  |  |
| 1. Drug use |  |  |  |  |  |
|  |  |  |  |  |  |
|  |  |  |  |  |  |
|  |  |  |  |  |  |
| **TOTAL: /24** |  |  |  |  |  |
| **Social Support and Motivation** | | | | | |
| 1. Social support |  |  |  |  |  |
|  |  |  |  |  |  |
|  |  |  |  |  |  |
|  |  |  |  |  |  |
| 1. Access to vehicle/transportation and medical care |  |  |  |  |  |
|  |  |  |  |  |  |
|  |  |  |  |  |  |
|  |  |  |  |  |  |
| 1. Coping |  |  |  |  |  |
|  |  |  |  |  |  |
|  |  |  |  |  |  |
|  |  |  |  |  |  |
| 1. Values/Motivation |  |  |  |  |  |
|  |  |  |  |  |  |
|  |  |  |  |  |  |
|  |  |  |  |  |  |
| **TOTAL: /12** |  |  |  |  |  |
| **Total SCORE:** | **/120** | **/120** | **/120** | **/120** |  |
| **List of Unacceptable/Unmitigable Items** | | | | | |
|  |  |  |  |  |  |
