# Supplementary material for: Implementation and clinical impact of an interdisciplinary tool to promote skin integrity after flap surgery in Veterans with spinal cord injury
Source: J Spinal Cord Med. 2024 Nov 20;48(3):415–28. doi: 10.1080/10790268.2024.2420434 (PMC12035953; doi:10.1080/10790268.2024.2420434)
Supplement: Supplement 1 - Flap Surgery Protocol.doc [file YSCM_A_2420434_SM1377.doc]

**Purpose of Protocol:** To provide guidance regarding perioperative and postoperative care for patients with spinal cord injury or disorder (SCI/D) receiving flap surgery for stage 4 pressure injury

**Wound admission for flap evaluation:**

- Prior to admission, the wound care team identifies patients with stage 4 pressure injury who may be candidates for flap surgery and consults with plastic surgery to further assess for this.
- The SCI/D interdisciplinary care team completes the Spinal Cord Optimization, Rehabilitation, and Empowerment (SCORE) tool before admission if possible, or otherwise upon admission.
- Assess for signs of wound infection; consider CT or MRI to evaluate for osteomyelitis or deep abscess. If initial surgical debridement is indicated, obtain deep cultures. Use topical wound antimicrobial (e.g., Dakin solution) to clear out nonviable tissue and colonization. Treat cellulitis with 5-14 days of antibiotics, and active osteomyelitis with 6 weeks of antibiotics, with at least 2 weeks completed prior to flap surgery. Consult infectious disease specialist if needed.
- Preoperative medical evaluation
- Labs needed: complete blood count (CBC), comprehensive metabolic panel, prealbumin, cystatin C, C-reactive protein (CRP), erythrocyte sedimentation rate (ESR); also obtain type and screen the day before surgery
  - Prealbumin must be ≥ 18 prior to surgery
- Verify patient has been nicotine-free for six weeks prior to flap surgery
- Feeding tube placement if recommended by team and patient agreeable
- Intravenous (IV) access prior to surgery; consider peripherally inserted central catheter (PICC) if appropriate
- Order specialty mattress (Immerse™ or Dolphin® mattress)
  - Physical therapy (PT) with wound, ostomy and continence nurse (WOCN) pressure maps patient on mattress to ensure good pressure redistribution and to determine best lying positions postoperatively

**Perioperative:**

- Nursing zero bed weight prior to surgery
- Hospital gown and Rooke® boots on patient
- Empty colostomy and urine bags
- Patient goes to operating room (OR) in hospital bed
- PICC line/IV access should be maintained until medically stable per primary team

**Week 1 (postop days 1-6):**

- Discuss timing of restart of anticoagulation with surgery team
- Strict bedrest, with head of bed (HOB) < 10⁰; okay to use reverse Trendelenburg position for comfort, meals, or tube feeding for limited periods
- No use of bed trapeze allowed
- No range of motion until postoperative day (POD) 8
- Positioning:
  - WOCN to assess for best positioning, using mechanical lifts for repositioning
  - Turns every two hours; assist patient with all turns
    - Avoid surgical side; avoid positioning pillows over incision
  - Completely relieve incisions, flap, and entire surgical area from pressure, friction, bending, or shear
  - Be careful to not pull, touch, or press on surgical area. Mark on patient the “do not touch” zone.
  - Position with Z-Flo™ fluidized positioners and pillows according to WOCN guidance
  - Full skin inspection every shift due to bedrest
- Flap surgery site:
  - Inspect flap incision every four hours; monitor for drainage, redness, firmness (should be soft), swelling, and signs of infection
  - Protect incision from trauma
  - Protect incision from contaminants, keeping incisions clean and dry (no barrier ointment)
  - Cleanse around Jackson-Pratt (JP) drains and apply drain sponge (change daily)
- Vacuum-assisted closure (VAC) therapy:
  - Incisional VAC dressing is applied in OR
  - Negative pressure runs at 125 mmHg on continuous mode
  - WOCN removes VAC dressing on POD 5-7
- JP drains:
  - Strip drains (with barrier ointment or alcohol pad) every hour
  - Empty drainage and document output and color every shift
  - Discontinue drain when output < 30 ml per 24 hours for two days
    - Pull one drain at a time, waiting at least two days after previous drain pulled
    - Pull drains at 14 days regardless of volume of drainage
- Intermittent pneumatic compression of legs for deep vein thrombosis prophylaxis
  - Remove on POD 21 when stretching starts
- Nutrition:
  - Registered dietitian (RD) to write orders for assisting with meal setup and feeding as needed
  - Medical provider to restart diet and tube feeding when medically appropriate; follow RD recommendation from last nutrition note
- Encourage incentive spirometry and cough/deep breathing every hour while awake

**Week 2 (postop days 7-13):**

- Continue with cares from week 1
- Okay to start upper body range of motion if needed
- POD 8: May initiate use of supine arm ergometer (except sacral and gracilis flaps) if approved by WOCN
- HOB can be elevated to 20⁰ for meals only, for 30 minutes three times a day (TID)
- Start application of barrier ointment to incisions four times a day
- Okay to start electrical stimulation therapy for incisional dehiscence as appropriate

**Week 3 (postop days 14-20):**

- Continue with cares from week 2
- All JP drains should be removed regardless of volume of output.
- Remove flap incision staples and sutures from stable part of incision; wait on areas that are pulling or unstable.
- POD 15: May initiate use of supine arm ergometer for sacral and gracilis flaps if approved by WOCN

**Week 4 (postop days 21-27):**

- Okay to increase HOB to 20⁰ if flap is stable
- Remove remainder of flap incision staples and sutures
- WOCN and PT co-treat to evaluate stretching and create bolster stretch program
- PT will direct stretching program and write patient-specific directions as nursing text order and printed schedule in Kardex and patient room.
- Use the foam bolster for stretching (low side or high side of bolster will be directed by PT).
- Begin at 15 minutes TID and increase by 15 minutes after three successful trials; repeat process until up to two hours TID based on patient needs.
- Monitor for signs of incisional dehiscence.

**Week 5 (postop days 28-34):**

- Begin sitting program under PT direction:
  - Pressure map wheelchair cushions before initiating sitting in wheelchair.
  - Transfer only with mechanical lift, using long seat mesh slings. Do not use removable Combi slings.
  - Perform skin inspection and tissue tolerance tests after each sitting episode.
    - Contact PT and/or WOCN with concerns.
  - Begin at 15 minutes TID and increase by 15 minutes after three successful trials; repeat process until up to two hours TID based on patient needs.
    - Bolster and sitting should always be paired back-to-back for total of two hours per session
  - Recommend lying down for minimum of 60 minutes after each sitting episode prior to getting up again.
  - During any sitting episode, patient must complete pressure relief (with tilt if power wheelchair is present) every 15 minutes for a minimum of two minutes.
- Once up to sitting two hours TID, reduce frequency of application of barrier ointment to flap incisions to twice a day (BID).

**Week 6 (postop days 35–41):**

- Begin transfer training, with special care to avoid shear or other skin/flap injury. Most patients will need to continue mechanical lift transfers to adequately protect the flap tissue.
- Occupational therapy to determine appropriate shower/commode chair, if not done already
- If patient is sitting two hours TID, okay to start showering as appropriate. Continue to use long seat mesh sling for all mechanical lift transfers.

**Week 7 (postop days 42-49):**

- Develop discharge instructions and plan of care
- Repeat interdisciplinary SCORE tool
- Photo of flap incision at time of discharge (WOCN)
- Consider feeding tube removal, if present. Depending on baseline aspiration risk, ability to maintain adequate oral intake, presence of malnutrition, and status of wound/flap, the patient will likely not need to keep the feeding tube in place after discharge.
  - If feeding tube recommended at discharge, RD to provide tube feeding education and order supplies

**Discharge follow-up plan:**

- Follow-up visits:
  - WOCN: two weeks after discharge, then monthly for one year
  - PT: two weeks after discharge, then monthly for six months, then at 12 months and as-needed
  - SCI/D medical provider and RD: as indicated
  - Check weights and labs during follow-up: CBC, CRP, ESR, prealbumin, basic metabolic panel
  - Photo of flap incision at 6 months after discharge
- Check flap incisions and apply barrier ointment to incisions BID for one year. Monitor for non-blanching erythema, dehiscence, and over-stretching.
- Monitor for signs and symptoms of infection of flap (e.g., puckering of tissue, firmness, redness).
  - If concerned, obtain photo, x-ray, and consultation with plastic surgery.
- Continue sitting limitation of 2 hours TID through week 14.
  - Starting at week 15, if flap is stable, may advance duration of sitting episode(s) by 30 minutes every three days as tolerated by skin until up to maximum of 6 hours at week 18. Closely monitor for flap concerns.
  - Recommend lying down for minimum of 30-60 minutes or fully tilting back in power wheelchair (if present) for 60 minutes after each sitting episode prior to getting up again.
  - During any sitting episode, patient must complete pressure relief (with tilt if power wheelchair is present) every 15 minutes for a minimum of two minutes. Time between pressure reliefs may be advanced to 30 minutes after one year if there are no skin/flap concerns.
- Check wheelchair cushion and other equipment daily to ensure proper functioning.

**Skin graft and donor site postoperative care:**

- Week 1:
  - Donor site: Cover with Tegaderm™ absorbent dressing for 2 weeks; change at one week
  - Graft site: Wound VAC dressing for one week
- Week 2:
  - Donor site: Change Tegaderm™ absorbent dressing for one more week
  - Graft site: Remove VAC dressing POD 5-7; apply Xeroform gauze daily for one week
- Week 3:
  - Donor site: Moisturize BID
  - Graft site: Moisturize BID

**Care for Integra® Dermal Regeneration Template:**

- Week 1: Wound VAC dressing for one week
- Weeks 2-3: Silicone layer will peel off; dressing changes with normal saline-soaked gauze BID
- Week 4 or after: Skin graft surgery
