# Supplementary material for: Implementation and clinical impact of an interdisciplinary tool to promote skin integrity after flap surgery in Veterans with spinal cord injury
Source: J Spinal Cord Med. 2024 Nov 20;48(3):415–28. doi: 10.1080/10790268.2024.2420434 (PMC12035953; doi:10.1080/10790268.2024.2420434)
Supplement: SCORE Supplementary material list.docx [file YSCM_A_2420434_SM1376.docx]

Supplementary material

Supplement 1: Flap Surgery Protocol of the Minneapolis VA Health Care System (MVAHCS) Spinal Cord Injury and Disorders (SCI/D) Center

Description: This is the updated version of the perioperative and postoperative care plan used by our center for flap surgeries in SCI/D patients. The postoperative care guidance includes flap site (and graft site, when applicable) care, patient positioning and handling, bedrest and progressive stretching/sitting protocol, pressure relief recommendations, and post-discharge follow-up needs. It was implemented in 2009 to help reduce postoperative complications, especially early complications.

Supplement 2: Minneapolis Spinal Cord Optimization, Rehabilitation, and Empowerment (SCORE) tool, updated version

Description: This is the updated version of the SCORE tool, which is considered a living document to remain relevant to current best practices. It includes additional scored items and much more guidance for scoring compared to the original 2012 version; it also provides suggestions for risk mitigation. The reason for more detail in this version is to more fully represent how our team uses SCORE and to reduce subjectivity in scoring items. Since it has more scored items, the original total score risk stratification no longer applies, but the primary focus of our team is now to support patients and caregivers in effective problem-solving to address specific concerns identified in SCORE.
